# Reply to ‘kidney transplantation in a patient with HIV disease’

**Published:** 2009-10

**Authors:** S. B. Bansal, M. Singhal, R. Ahlawat, V. Kher

**Affiliations:** Department of Nephrology and Kidney Transplantation, Fortis Hospital, Noida, India

Sir,

I thank Dr. Gonzalez for replying.[1] I want to make following points in response:

1. The goal of initial HAART in the naïve patient should be to devise a regimen that will achieve maximal durable viral suppression (<50 copies/mL) and be tolerated for an indefinite period of time.[2,3]
2. For ARV therapy-naïve patients, the initial HAART regimen should include a combination of two nucleoside reverse transcriptase inhibitors (NRTIs) plus either a ritonavir-boosted protease inhibitor (PI), or a non-nucleoside reverse transcriptase inhibitor (NNRTI). Unfortunately, there is no clear data available at present on which to base distinction between these two approaches.[2,3]
3. The use of NNRTI-based regimen for initial therapy may preserve PIs for later use.[3] For NNRTI the preferred drug is efavirenz, but nevirapine can be used as an alternative drug in combination with two NRTIs.[3]
4. We agree with you that clinicians should not use nevirapine as part of the initial regimen in women with CD4 counts >250 cells/mm^3^ or men with CD4 counts >400 cells/mm^3^. However, it can be used in men and women with CD4 counts <400 and <250 cells/mm^3^, respectively.[3] As this patient was already on this regimen, when he came to us for transplant and was responding to treatment as evident by his CD4 cell count and HIV RNA levels.[4]
5. The nevirapine as initial treatment is contraindicated only in cases of moderate to severe hepatic impairment (Child-Pugh score B or C).[3] This patient had normal hepatic function initially and later on he had mild increase in aminotransferase levels, which improved after reduction in dose of drug.[4]
6. When nevirapine or efavirenz were used as initial treatment, no significant difference was noted in efficacy between these two drugs in combination with stavudine and lamivudine and toxicity was only slightly higher with nevirapine.[5]
7. Lamivudine/stavudine/nevirapine is one of the prescribed regimens for treatment of HIV in African countries as approved by the World Health Organization. It is used as a first-line combination in eight African countries. This is also the most cost-effective initial drug regimen.[6]
8. I agree with the author that measuring serum antiretroviral drug levels after transplant might help with drug dosing.
